# Valedictory

**Published:** 1881-09

**Authors:** Harvey L. Byrd


VALEDICTORY.—Since the removal of this journal to New York city the proprietor has found it to his interest to make some changes in its management, and as further continuance with it would be incompatible with other duties, present and prospective, in Baltimore, I must part with my indulgent readers with emotions amounting to real sorrow.

Meanwhile, I ask all to support the INDEPENDENT PRACTITIONER in the great metropolis, and beg to tender to each and to all my warm and sincere wishes for their continued prosperity and happiness. HARVEY L. BYRD.

